# Constraints on coastal dune invasion for a notorious plant invader

**DOI:** 10.1093/aobpla/plv126

**Published:** 2015-11-11

**Authors:** Alden B. Griffith, Tania Ahmed, Abigail L. G. Hildner, Shivani Kuckreja, Shuangxou Long

**Affiliations:** Environmental Studies Program, Wellesley College, Wellesley, MA 02481, USA

**Keywords:** *Bromus tectorum*, Cape Cod, cheatgrass, disturbance, invasion, population dynamics, seed dispersal

## Abstract

Although most biological invasions are not successful, relatively few studies have examined otherwise notorious invaders in systems where they are not highly problematic. The annual grass *Bromus tectorum* is a dominant invader in western North America, but is usually confined to human-dominated and disturbed systems (e.g. roadsides and parking lots) in the East where it remains virtually unstudied. This study aims to address fundamental ecological questions regarding *B. tectorum* in a Cape Cod dune ecosystem. (i) What is the range of variation in population dynamics and the potential for population growth? (ii) Which factors influence its local abundance and distribution? We observed substantial variation in population dynamics over 3 years, with the number of adult *B. tectorum* individuals increasing substantially between the first 2 years (λ = 9.24) and then decreasing (λ = 0.43). Population growth in terms of total seeds was similarly variable, but to a lesser extent (λ = 2.32 followed by λ = 0.32). Experimental soil disturbance led to a more than 10-fold increase in mean seedling emergence, and high sensitivity to differences in emergence carried this effect through the life cycle. In contrast, barriers to seed dispersal had no effect on population dynamics, suggesting limited dispersal in this system. Across the landscape, the presence of *B. tectorum* was associated with areas of higher plant diversity as opposed to those with a strong dominant (e.g. the foredune, dominated by *Ammophila breviligulata,* or low heathlands, characterized by *Hudsonia tomentosa* and *Arctostaphylos uva-ursi*). Overall, we find that *B. tectorum* is capable of both substantial population growth and decline in a dune ecosystem, but is likely limited without disturbance and dispersal agents. Thus, management actions that restrict dune access (e.g. for nesting habitat) likely have the co-benefit of limiting the invasive potential of *B. tectorum*.

## Abstract (precis)

Nonnative invasive species can be highly problematic in some ecosystems, but much less so in others. For example, the grass *Bromus tectorum* is a notorious invader in western North America, but it remains virtually unstudied in the East where its overall impact has been lower. We examined fundamental ecological questions of the distribution and abundance of *B. tectorum* in a coastal dune system on Cape Cod and found annual population growth to be highly variable (e.g. capable of ‘booms’ and ‘busts’). However, our results suggest that limitations to disturbance and seed dispersal likely constrain invasion in this ecosystem.

## Introduction

Nonnative species often face a broad range of ecological challenges and constraints in novel environments, which results in a high rate of failure for species introductions (Williamson 1996; Mack *et al*. 2000). For species that manage to become established and naturalized outside of their native range, only a small fraction tend to become highly invasive with significant ecological or economic impacts (Davis 2009; Simberloff *et al*. 2013). Similarly, there can be high variability in invasive success within a species, with naturalized populations exhibiting low rates of spread and/or impact in one region, but rapid spread and negative impacts on another (e.g. Richardson and Rejmánek 2004). Although of high relevance in general to invasion biology, relatively few studies have examined otherwise notorious invaders in systems where they are not highly problematic (Zenni and Nuñez 2013).

*Bromus tectorum* is an annual grass native to Europe and Eurasia that has become invasive outside of its native range, particularly in western North America. Here it has come to dominate and transform millions of hectares of arid and semi-arid ecosystems in the Intermountain West (Mack 1981; Knapp 1996). It has thus unsurprisingly been the focus of a substantial amount of research in this region over many decades. However, *B. tectorum* is naturalized in every state in the USA and in all Canadian provinces other than Newfoundland (USDA 2015). Moreover, it was introduced to eastern North America before spreading to the West (Novak and Mack 2001). Yet in the eastern half of the continent, *B. tectorum* has generally neither been aggressively invasive nor problematic. This is mirrored by little research on the species in the East. Of the 903 articles returned from a *Web of Science Core Collection* search of the term ‘Bromus tectorum’, only three appear to be specifically focussed on *B. tectorum* in eastern North America. All three studies examined large-scale patterns of genetic variation and invasion history (Bartlett *et al*. 2002; Valliant *et al*. 2007; Huttanus *et al*. 2011). To our knowledge, there has been no published study investigating *in situ* ecological dynamics of *B. tectorum* in eastern North America.

In the East, *B. tectorum* is almost always found in human-dominated and disturbed systems (A. B. Griffith, pers. obs.). Indeed, Bartlett *et al*. (2002) wrote that they collected tissue samples from populations in the eastern USA that were ‘found in disturbed habitats near roadsides, railroad tracks, abandoned fields and construction sites.’ In contrast, the research presented here was initially motivated by the observation of *B. tectorum* growing in coastal dune ecosystems in Massachusetts, occasionally in the absence of any obvious disturbance. These observations of *B. tectorum* in dune communities are supported by recent large-scale vegetation surveys that find *B. tectorum* to be occasional in coastal communities in southern New England and Cape Cod (Smith 2006; Von Holle and Motzkin 2007).

Beyond its mere presence in coastal dunes, there are compelling reasons to examine the ecology of *B. tectorum* in these ecosystems. First, although coastal dune ecosystems account for a relatively small amount of land area (∼0.5 % in Massachusetts), they represent specific and/or critical habitat for many species and often support high levels of ecological diversity (Martínez *et al*. 2008). Moreover, dune systems provide important ecosystem services as buffers of the dynamic land/sea interface (Barbier *et al*. 2011; Calvão *et al*. 2013). Finally, compared with other ecosystems in the East, dune systems have several characteristics that are similar to ecosystems in the Intermountain West that *B. tectorum* has invaded so successfully: these systems tend to share relatively infertile and sandy soils, exhibit extremes in soil surface temperature, favour bunchgrasses over sod-forming grasses and typically have relatively low vegetative cover and overall productivity.

Yet *B. tectorum* is not entirely new to dune systems and was described a century ago by Bicknell (1918) on Nantucket:

Up to 1908 this grass had become established only sparingly, although growing freely on the low dunes near the bathing beach and observed at stations as far east as Polpis and Pocomo and west towards Madequet. In succeeding years it was found to be spreading freely and fast becoming common*.*

At the time of Bicknell's publication, *B. tectorum* was beginning to spread rapidly in the West and would become problematic over a vast range in a matter of decades (Mack 1981). The same outcome has not come to pass in East Coast dune systems, although the grass was found to be dominant under invasive black locust trees (*Robinia pseudoacacia*) in the Indiana Dunes (Peloquin and Hiebert 1999). Such different outcomes likely depend on the intersection of traits specific to *B. tectorum*, the characteristics of particular ecosystems and patterns of land management and land use history (Theoharides and Dukes 2007; Bradley *et al*. 2010; Vilà and Ibáñez 2011).

With the substantial body of research in the western USA and the general lack of investigation in the East, this study aims to address fundamental ecological questions regarding *B. tectorum* in a coastal dune ecosystem: (i) what is the range of variation in population dynamics and the potential for population growth? (ii) which factors influence its local abundance and distribution? Specifically, we focus on aspects of seed dispersal, soil disturbance, plant community composition, nitrogen (N) availability and interannual climatic variation. These questions are situated within larger questions of invasion potential and the differences between driving factors of *B. tectorum* invasion in western versus eastern North America.

## Methods

### Site description

The study site is located between the foredune and coastal bluff adjacent to Cape Cod Bay in Wellfleet, MA (41.943°N 70.075°W). The area is protected as part of the Cape Cod National Seashore, although there is some evidence of previous human disturbance (e.g. an old trail that leads down from the bluff). Natural disturbance from high tide storm events appears to be occasional but limited, with wrack deposits found in some sampling plots.

The site spans the vegetation transition from the foredune (dominated by *Ammophila breviligulata*) to low heath plant communities (characterized by *Hudsonia tomentosa* and *Arctostaphylos uva-ursi*). Soils consist mostly of sand with relatively little organic matter or humus. The most common vascular plant species are *A. breviligulata*, *H. tomentosa*, *Artemisia campestris* and *Deschampsia flexuosa*. Lichens (*Cladonia* spp.) are extremely common across the entire site. *Bromus tectorum* is found in patches throughout much of the site and was documented at this location by Smith (2006).

### Sampling plots

In 2012, we established 18 plots (0.25 m^2^ each) to monitor *B. tectorum* population dynamics (hereafter ‘demographic plots’). The plots were arranged into three blocks of six plots each. As the plots were intended to track population dynamics, their overall locations within the research site were not random, but were determined by the presence of *B. tectorum*. Within each block location, individual plots were arranged in a 3 × 2 grid (each 1 m apart), and thus their specific locations were not targeted. The following year (2013) we established three 100 m transects that run parallel to the shore (7 m apart) with 0.25 m^2^ plots every 5 m (63 total plots; hereafter ‘community plots’). These plots complement the demographic plots by allowing for a broader examination of *B. tectorum* spatial patterns and biotic/abiotic associations.

### Population sampling

Each June from 2012 to 2014, we recorded the number of *B. tectorum* individuals within demographic plots and counted the number of spikelets per individual. Plants were typically senesced at this point and missing spikelets were included in the tally by noting bare glumes (*B. tectorum* appears to have a relatively short life cycle at this site, with seedling establishment typically in April and seed set in June/July). Mean individual plant fecundity was estimated using a mean value of 1.7 (SD = 0.76) seeds per spikelet as determined by off-plot sampling of over 120 spikelets. From these data, two metrics of the population growth rate (λ) were assessed based on different annual transition periods in the life cycle: seeds to seeds (λ_seeds_) and adult plants to adult plants (λ_adults_). The combined rates of seedling establishment and survival for each plot were estimated by dividing the number of adults by the number of seeds produced the previous year. This estimate assumes local seed dispersal, which is supported by year-to-year plot consistency **[see Supporting Information—Fig. S1]** and results from the seeding experiment (detailed below).

### Seeding experiment

We performed an experiment in order to constrain values of seedling emergence, examine the potential for seed dispersal, and investigate the importance of small-scale disturbance/seed burial. In early November 2012, locally collected seeds of *B. tectorum* were scattered over small plots (∼15 × 20 cm), situated in areas several metres away from existing *B. tectorum* individuals. The potential for seed dispersal was manipulated by leaving plot edges open or by enclosing the plots with a wooden frame (∼2 cm tall) covered by a coarse mesh screen. Within each dispersal treatment, seeds were either scattered directly onto the surface or were gently raked into the sand with several motions of a hand rake. This represents a moderate level of disturbance, resulting in slight-to-incomplete seed burial. It was intended to be similar to the impact from limited sand redistribution or mammal/human movement. Each plot contained 100 seeds, and the experiment was replicated in four complete blocks spaced throughout the research site. Emerging seedlings were counted on 15 April 2013 and surviving adult plants and total spikelet production were assessed on June 2013. The effects of the dispersal barrier and disturbance treatments along with their interactions were examined with an analysis of variance (ANOVA) using log-transformed data (JMP 11, SAS Institute, Cary, NC, USA).

To better understand the effect of the disturbance treatment, we created a simple periodic population model, with single life history stages existing at different times of the year: seeds, seedlings and adult plants. The overall model can be represented as, λ_seeds_ = emergence × survival × fecundity. These transition values were determined as the mean of plot-level values, and variability was assessed using bias-corrected confidence intervals (Caswell 2001). We used this simple model and a Life Table Response Experiment (LTRE) analysis to examine how effects on seedling emergence translate into population-level effects (Caswell 2001) **[see Supporting Information—Table S1]**.

### Community sampling and analysis

In 2013, we characterized the vegetation within the community plots. The cover for all vascular plant species and lichen within each plot was assessed on a scale from 0 to 8 that approximates percent cover: each plot was sectioned into quarters and the cover of each species was scored from 0 (absent) to 2 (covering most of quarter). Plot values were then the sum of the quarter scores. *Bromus tectorum* within the plots was sampled as in the demographic plots (above).

Plot-level similarity in the plant community (excluding *B. tectorum*) was examined using a non-metric multidimensional scaling (MDS) analysis based on Bray–Curtis dissimilarity values. We tested for differences in background community similarity between plots with and without *B. tectorum* using an analysis of similarities (ANOSIM). Based on observations from the MDS analysis, we tested for relationships between *B. tectorum* presence/absence and the cover of common plant species, (e.g. *A. breviligulata*, *H. tomentosa*, *A. campestris*, *D. flexulosa*, *A. uva-ursi*) using logistic regression. We also quantified plot-level diversity of the plant community (excluding *B. tectorum*) using the Shannon–Wiener index and related this to the presence of *B. tectorum*. All community analyses were performed in R (R Core Team 2014). The MDS and ANOSIM analyses were performed using the ‘vegan’ package (Oksanen *et al*. 2013) and logistic regressions were performed using the ‘brglm’ package (Kosmidis 2013).

In order to examine potential mechanisms behind observed spatial associations, we tested for soil mineral N levels in 2014. *Bromus tectorum* has been shown to respond strongly to soil N (Lowe *et al*. 2003), and its spatial associations with other species may be influenced by abiotic differences associated with microhabitat (Griffith and Loik 2010). Samples were collected adjacent to each community plot (15–20 cm depth) and were dried in a forced-air oven at 45 °C for 96 h. Analyte extracts were prepared by adding 7 g of dry and homogenized soil to 40 mL of 2 M KCl solution. Extracts were shaken for 1 h, allowed to settle for an additional hour, and were then filtered. The concentrations of ammonium $\left(\text{N}{{\text{H}}_{4}}^{+}\right)$ and nitrate $\left(\text{N}{{\text{O}}_{3}}^{-}\right)$ were measured by colorimetric analysis (two replicates per sample) using an Astoria-Pacific Discrete Analyzer (Astoria-Pacific, Inc., Clackamas, OR, USA). Potential effects of soil N on *B. tectorum* were examined by relating plot-level nitrate, ammonium and total mineral N levels to *B. tectorum* presence as well as the number individuals, total seed production and mean individual seed production (for plots that contained *B. tectorum*).

## Results

### Population dynamics

**Figure 1.**
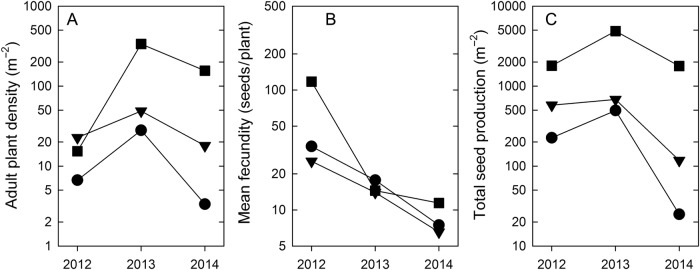
*Bromus tectorum* population dynamics between 2012 and 2014. Values for (A) adult plant density and (C) total seed production are the sums of plot-level data for each of the three demographic sampling areas. (B) Fecundity values were calculated from the sums of plant and seed production density. (Sums were used instead of means due to zero values for some plots, and thus represent the combined area of six sampling plots). Symbols refer to spatially distinct sampling areas.

Overall, *B. tectorum* population growth and underlying metrics were highly variable between 2012 and 2014 (Fig. 1). There was a nearly 10-fold increase in the density of adult individuals between 2012 and 2013 (Table 1; λ_adults_ = 9.24), accompanied by more than a 2-fold increase in seed production (λ_seeds_ = 2.32). This was the product of relatively high individual fecundity in 2012 (58.3 seeds per plant) followed by a high fraction of seeds becoming adults in 2013 (15.9 %, assuming local dispersal and no seedbank). Despite comparatively low fecundity values in 2013 (14.7 seeds per plant), the large adult population resulted in high total seed production.

**Table 1.** Population-level transition values between years. Annual growth rates (λ) are based on both changes in total adult plants and total seed production. The establishment and survival rates was estimated from the ratio of adult plants to seed production the previous year. Values are based on totals across all demographic plots.

	2012–13	2013–14
Adult–adult annual growth rate, λ_adults_	9.24	0.43
Seed–seed annual growth rate, λ_seeds_	2.32	0.32
Establishment and survival (%)	15.9	2.93

Between 2013 and 2014, the number of adults was roughly halved (Table 1; λ_adults_ = 0.43) with even greater decreases in total seed production (λ_seeds_ = 0.32). This decrease was primarily associated with a much reduced fraction of seeds becoming adults in 2014 (2.9 %). Overall, individual fecundities and adult densities compensated each other to some extent in 2012 and 2013, but both values decreased in 2014 (Fig. 1).

### Seeding experiment

**Figure 2.**
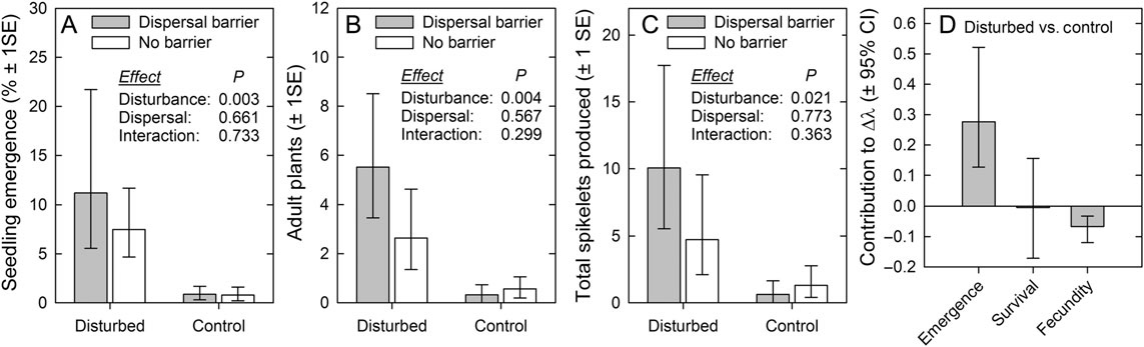
Results of the 2012/2013 seeding experiment. (A) Seedling emergence in April, (B) total adult plants in June and (C) total spikelets produced (values are back-transformed means ± 1 SE). (D) The LTRE analysis indicates how differences in life cycle transition values between the disturbed and control treatments contributed to differences in population growth rates (contributions to Δλ ± 95 % CI).

Moderate disturbance, as implemented by manually raking seeds into the soil, was associated with a >10-fold increase in *B. tectorum* seedling emergence in April 2013 (Fig. 2A; *F*_1,12_ = 14.4, *P* = 0.003). This effect carried through the lifecycle with increased numbers of adult plants (Fig. 2B; *F*_1,12_ = 12.3, *P* = 0.004) and total plot spikelet production in June (Fig. 2C; *F*_1,12_ = 7.07, *P* = 0.023). Restricting seed movement with dispersal barriers did not significantly increase the number of seedlings emerging within the plots (Fig. 2A; *F*_1,12_ = 0.202, *P* = 0.661) nor was there any interaction with soil disturbance (Fig. 2A; *F*_1,12_ = 0.122, *P* = 0.732). This result is paired with observations of ungerminated seeds remaining within the plots that were open to dispersal. There remained no significant effects of the dispersal barrier treatment nor its interaction with the disturbance treatment throughout the rest of the growing season.

Our simple population model indicated a higher population growth rate associated with the disturbance treatment (λ_seeds_ = 0.25) compared with the control (λ_seeds_ = 0.04). (Note that these values are much lower than the population growth rate observed on the demographic plots that year, likely indicating lower microsite suitability compared with naturally occurring populations.) Population growth rates in the experimental plots were most sensitive to changes in seedling emergence rates, with emergence sensitivity values 4.2 and 43 times greater than seedling-to-adult survival sensitivity values for disturbed and control plots respectively **[see Supporting Information—Table S1]**. The LTRE analysis indicated that the observed increase in seedling emergence in the disturbed plots contributed almost entirely to the associated increase in the population growth rate (Fig. 2D).

### Community patterns

**Figure 3.**
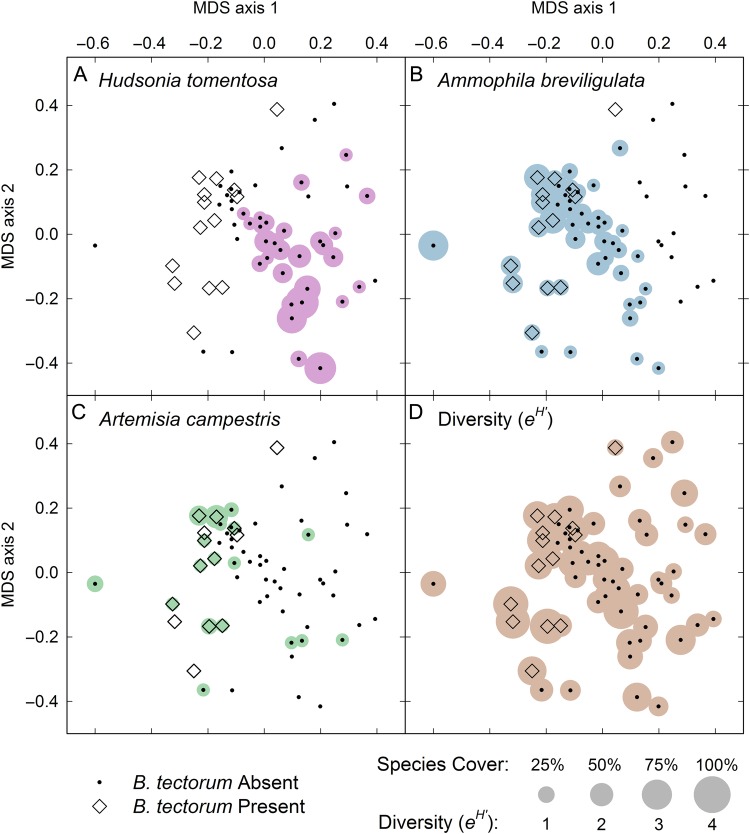
Multidimensional scaling analysis of plot-level plant community similarity in 2013 (excluding *B. tectorum*). Plots with and without *B. tectorum* are indicated and show significant separation (ANOSIM *P* = 0.03). The cover of common species (A–C) and overall plant diversity (D) within each plot is superimposed on the MDS.

**Figure 4.**
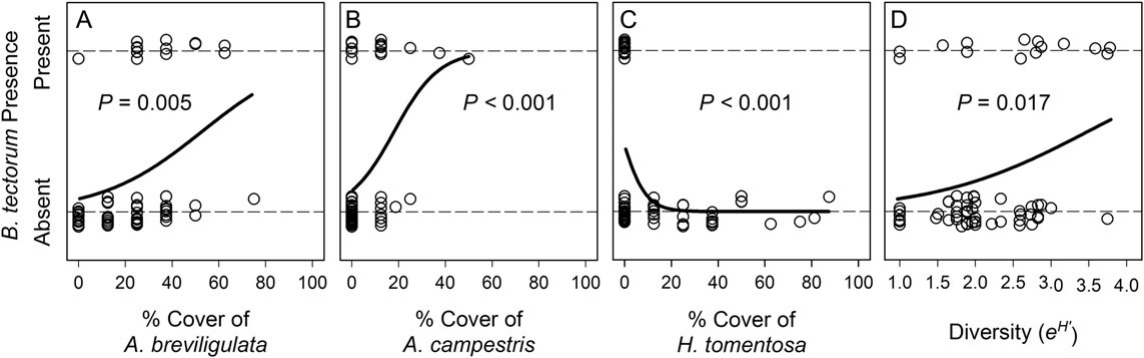
Logistic regressions relating *B. tectorum* presence to (A–C) the cover of particular species and (D) overall plant diversity. Presence/absence axis values are jittered to minimize visual overlap of points.

The MDS analysis identified a clustering of plots containing *B. tectorum* based on similarity in plant community composition (stress = 0.18; Fig. 3). An associated ANOSIM revealed a significant difference in the plant community for plots with and without *B. tectorum* (*R* = 0.15, *P* = 0.03). Specifically, *B. tectorum* was absent in all plots that contained *H. tomentosa*, whereas the probability of finding *B. tectorum* increased with increasing cover of *A. breviligulata* and *A. campestris* (Fig. 4A–C). When considering all three of these species as predictor variables for *B. tectorum* presence, the most parsimonious model (lowest AIC score) included only *H. tomentosa* and *A. campestris*.

The presence of *B. tectorum* was also associated with higher plant diversity, as assessed by the Shannon–Wiener index (Fig. 4D). Additionally, mean individual fecundity for *B. tectorum* in the community sampling plots in 2013 was negatively associated with overall vegetation cover (*r*^2^ = 0.34, *P* = 0.029; data not shown).

### Soil N

Levels of soil N were not found to be significantly related to the presence or abundance of *B. tectorum*. This was the case both when examined as single independent variables and when included in models with other predictors such as *H. tomentosa* and *A. campestris*. The strongest relationship to emerge was a non-significant positive trend between soil nitrate and the density of *B. tectorum* individuals (*r*^2^ = 0.22, *P* = 0.121).

## Discussion

### Population dynamics

The large variability across years in the number of *B. tectorum* individuals and seeds produced is not entirely surprising for an annual plant (Morris *et al*. 2008; Griffith and Loik 2010). However, the nearly 10-fold increase in the number of adult plants between 2012 and 2013 is substantial and demonstrates the capacity for population ‘booms’ of *B. tectorum* in dune systems. However, the growth rate with respect to adult individuals alone does not necessarily translate into changes in total cover or biomass, which may be much more relevant metrics from a management standpoint. Indeed, the relatively large population in 2013 consisted of small individuals on average, with a greatly reduced mean fecundity. Thus, the seed-to-seed population growth rate may be a more broadly useful metric, as it implicitly integrates both total adult plants and individual plant size. With this metric, variability remains high, with the overall population more than doubling between 2012 and 2013 and then reducing by two-thirds.

Drivers of temporal variation in population dynamics and the frequency of ‘boom’ and ‘bust’ years are clearly difficult to resolve without long-term study. However, variation in some metrics is more related to specific abiotic factors than it is to others (e.g. plant size integrates factors across the growing season), whereas establishment rates and the total number of individuals are more influenced by factors operating earlier in the life cycle. Given this broad framework, an initial hypothesis for the high rate of population growth between 2012 and 2013 is that favourable conditions during the 2012 growing season resulted in relatively fecund individuals (Fig. 1B), which was then followed by favourable conditions in early 2013 leading to relatively high establishment (Table 1). An analysis of nearby meteorological conditions **[see Supporting Information—Table S2, Fig. S2]** suggests that the most noteworthy aspect of 2012 was the ‘length’ of the growing season as opposed to mean conditions. There was hardly any snow cover throughout the 2011/2012 winter, and the accumulation of degree days experienced at the soil surface was advanced in 2012 by roughly a month compared with 2013 and nearly 2 months compared with 2014. Accumulated degree days has been shown to strongly predict germination timing for *B. tectorum* (Roundy *et al*. 2007), and early-germinating cohorts can have greatly increased fecundities (Mack and Pyke 1983). Our data, although limited to just 3 years, agree in that yearly rankings of degree days are aligned with rankings of mean fecundity. In contrast to the interpretation for high fecundities in 2012, there is no clear evidence that explains the relatively high rates of establishment and survival inferred for 2013 compared with 2014. Variation in seed dormancy remains a possibility. Observed rates of secondary seed dormancy for *B. tectorum* in the Western USA ranges from 0 to 30 %, but carryover beyond 2 years appears very low (Meyer *et al*. 2007). Also, high-end values for dormancy are associated with seeds produced in the autumn (with limited after-ripening), which is not the case here.

### Seeding experiment

Variation in population dynamics may also be driven by singular, episodic and/or small-scale events rather than by variation in mean or cumulative measures. Of particular relevance in dune systems is disturbance via sand redistribution, which can have important effects on germination and seedling emergence (Maun 1998). For *B. tectorum*, soil disturbance caused by grazing can promote establishment and likely played an important role in its invasion across the Western USA (Knapp 1996; Ponzetti *et al*. 2007; Reisner *et al*. 2013). In our study, moderate soil (sand) disturbance had a strong impact on *B. tectorum* seedling emergence, with rates 10-fold greater than in control plots where seeds were simply scattered on the surface. This effect strongly contributed to increased population growth due to the high sensitivity to changes in seedling emergence. Disturbance has long been associated with plant invasions (Theoharides and Dukes 2007), and several studies have demonstrated its relevance to the invasion of coastal dune systems in particular (Kim 2005; Marchante *et al*. 2010; Burkitt and Wootton 2011). (Although ironically, some invaders, such as *Ammophila* spp., can stabilize dunes and thereby ultimately reduce disturbance, e.g. Hacker *et al*. 2012). In contrast to moderate disturbance, extreme sand redistribution may result in deep burial, which is likely detrimental to *B. tectorum* populations considering its short-lived seedbank. (Such a case was observed at a second initial study location on the Atlantic coast of Cape Cod, where demographic monitoring was precluded due to plots becoming entirely covered and obscured by sand movement.)

Dispersal is another aspect of seed ecology that is highly relevant to invasions. Our experiment provided evidence for limited dispersal at this site, as restricting seed movement had no effect on local emergence (Fig. 2A). Additionally, the number of adult plants within plots was well-predicted by the number of seeds produced in the previous year **[see Supporting Information—Fig. S1]**, suggesting a local retention of seeds. These findings agree with previous studies of *B. tectorum* seed dispersal in the Western USA, where dispersal distances were found to be very limited (maximum <0.5 m) in vegetated areas (Kelrick 1991), but slightly higher in bare-soil areas (Johnston 2011).

Although a high dispersal capability can be associated with invasive success (Kolar and Lodge 2001; Sakai *et al*. 2001), limited dispersal may also be particularly important for annual plants to maintain fecund populations in suitable microhabitat in the absence of perenniating adults. Indeed, rapid spread across the landscape can be achieved by mostly local dispersal combined with small, but non-zero, probabilities of long-distance dispersal (e.g. ‘fat tailed’ dispersal kernels; Clark *et al*. 1998; Levin *et al*. 2003). Long-distance dispersal of *B. tectorum* in this system would likely necessitate specific vectors, such as vertebrates or humans. The overall results of our experiment align with observations of *B. tectorum* populations in Cape Cod dune systems growing in disturbed areas with obvious dispersal vectors, such as parking lot and road edges and along trails leading to beaches.

### Community patterns

Within our study location, the presence of *B. tectorum* was predictable to an extent based on the background plant community composition. Plots containing species more typical of low heathlands and ‘dune mats’ (e.g. the prostrate shrubs *H. tomentosa* and *A. uva-ursi*) were devoid of *B. tectorum*, whereas it was more commonly found in higher diversity communities closer to the foredune. It is important to note that, although we detected a significant relationship between increasing *A. breviligulata* cover and the presence of *B. tectorum*, extrapolation of this relationship to foredune systems dominated by *A. breviligulata* is not warranted: although not quantitatively sampled, *B. tectorum* was not observed on the foredune. This is consistent with Cheplick (2005) who found that annual plants tended to be less abundant in areas dominated by *A. breviligulata.* Rather, increasing cover of *A. breviligulata* in this case points to a transition zone immediately behind the foredune where many species, including *B. tectorum*, are more common. Of the 18 vascular plant species recorded in our plots, we found *A. campestris* to be the best single ‘indicator species’ for the presence of *B. tectorum*. Given the seeding experiment results above, it is important to consider whether the observed community associations could be an artifact of limited seed dispersal. Although there was one large area where *B. tectorum* was relatively abundant and *H. tomentosa* absent, *B. tectorum* was found throughout the entire site, with plots containing *B. tectorum* interspersed with plots containing *H. tomentosa* (five separate locations had adjacent plots containing *B. tectorum* and *H. tomentosa*). Thus, the alternative hypothesis that *B. tectorum* has yet to disperse into areas containing *H. tomentosa* is not well supported.

The fact that plots with high diversity were more likely to contain *B. tectorum* may highlight that factors promoting the presence of other non-dominant species also apply to *B. tectorum* and/or point to the significance of competitive dominants in dune systems. For plots that contained *B. tectorum*, there was some evidence of local competitive effects, with reduced fecundity in plots with high vegetation cover. However, soil N did not emerge as a significant factor, although the low range of values (mean inorganic *N* = 3.4 mg kg^−1^ ± 1.5) and the dynamic nature of N pools in well-drained sandy soils may make it difficult to resolve relationships.

Similar to the general community patterns observed here, Kim (2005) found that nonnative species in Korean sand dunes tended to proliferate at the transition zone with more inland ecosystems. If our observed community associations for *B. tectorum* apply more broadly to East Coast dune systems in general, then the total area conducive to invasion may be relatively limited. At the same time, it is of potential concern because of the association with communities of higher plant diversity. Although we did not specifically focus on the impacts of *B. tectorum* on dune communities, the overall densities in the community plots (mean of 8.2 adults m^−2^ and 114 seeds m^−2^ in 2013) remain low, although localized densities can be relatively high (Fig. 1A). These values are far below levels of invasive dominance observed in the Western USA, where mean seed production values can exceed 10 000 m^−2^ (Young *et al*. 1969; Smith *et al*. 2008).

## Conclusions

Although *B. tectorum* is generally widely distributed across Cape Cod at a coarse scale, it remains relatively uncommon in undisturbed dune systems (Smith 2006, A. B. Griffith, pers. obs.). Within a dune community, the local presence of *B. tectorum* appears to be nonrandom and is associated with areas of higher plant diversity, which are not strongly dominated by *A. breviligulata* or low heathland species. From a population perspective, *B. tectorum* is capable of both rapid growth and decline in a natural and relatively undisturbed dune system. With respect to invasion potential, such variability may be of concern as the chance event of several ‘boom’ years in a row could lead to rapid changes to the ecosystem. However, our data also suggest that seed dispersal in the absence of specific vectors is low, and therefore that invasion potential away from roads and trails may be limited. Moreover, important dispersal vectors for *B. tectorum* (e.g. humans and mammals) are also agents of disturbance, which our results suggest would directly facilitate population establishment in this system. The widespread combination of simultaneous dispersal and disturbance agents that played an important role in the invasion of *B. tectorum* in the Western USA (Mack 1981; Knapp 1996) appears to be much more limited in Cape Cod. This highlights the potential co-benefits of existing conservation policies in dune systems. For example, restricting access to dune areas in order to protect nesting habitat is also likely effective at reducing the risk of establishment and spread by *B. tectorum*. Overall, while *B. tectorum* appears to be able to successfully naturalize in dune systems, its potential for aggressive invasion may currently be limited.

## Sources of Funding

This work was supported by funding from Wellesley College, the Wellesley College Science Center Summer Research Program, and a Brachman Hoffman Small Grant.

## Contribution by the Authors

A.B.G. conceived the project, collected and analyzed data and drafted the manuscript. T.A., A.L.G.H., S.K. and S.L. collected and analyzed data, and outlined and edited the manuscript.

## Conflict of Interest Statement

None declared.

## Supporting Information

The following additional information is available in the online version of this article –

**Figure S1.** Adult plants related to local seed production the previous year.

**Table S1**. LTRE analysis.

**Table S2.** Accumulated degree days experienced by seeds.

**Figure S2.** Winter and growing season daily precipitation, snow depth and cumulative degree days.

Additional Information
