# Supplementary material for: Constraints on coastal dune invasion for a notorious plant invader
Source: AoB Plants. 2015 Nov 11;7:plv126. doi: 10.1093/aobpla/plv126 (PMC4676798; doi:10.1093/aobpla/plv126)
Supplement: Additional Information [file supp_7_plv126_index.html]

Constraints on coastal dune invasion for a notorious plant invader — Constraints on coastal dune invasion for a notorious plant invader — Additional Information 

# Constraints on coastal dune invasion for a notorious plant invader

## Additional Information

Additional Information

- Additional Information - Docx file
