# Supplementary material for: Constraints on coastal dune invasion for a notorious plant invader
Source: AoB Plants. 2015 Nov 11;7:plv126. doi: 10.1093/aobpla/plv126 (PMC4676798; doi:10.1093/aobpla/plv126)
Supplement: Additional Information [file supp_plv126_plv126supp.docx]

**SUPPORTING INFORMATION**

**Figure S1.** The number of adult *B. tectorum* individuals in each demographic plot related to local seed production the preceding year.


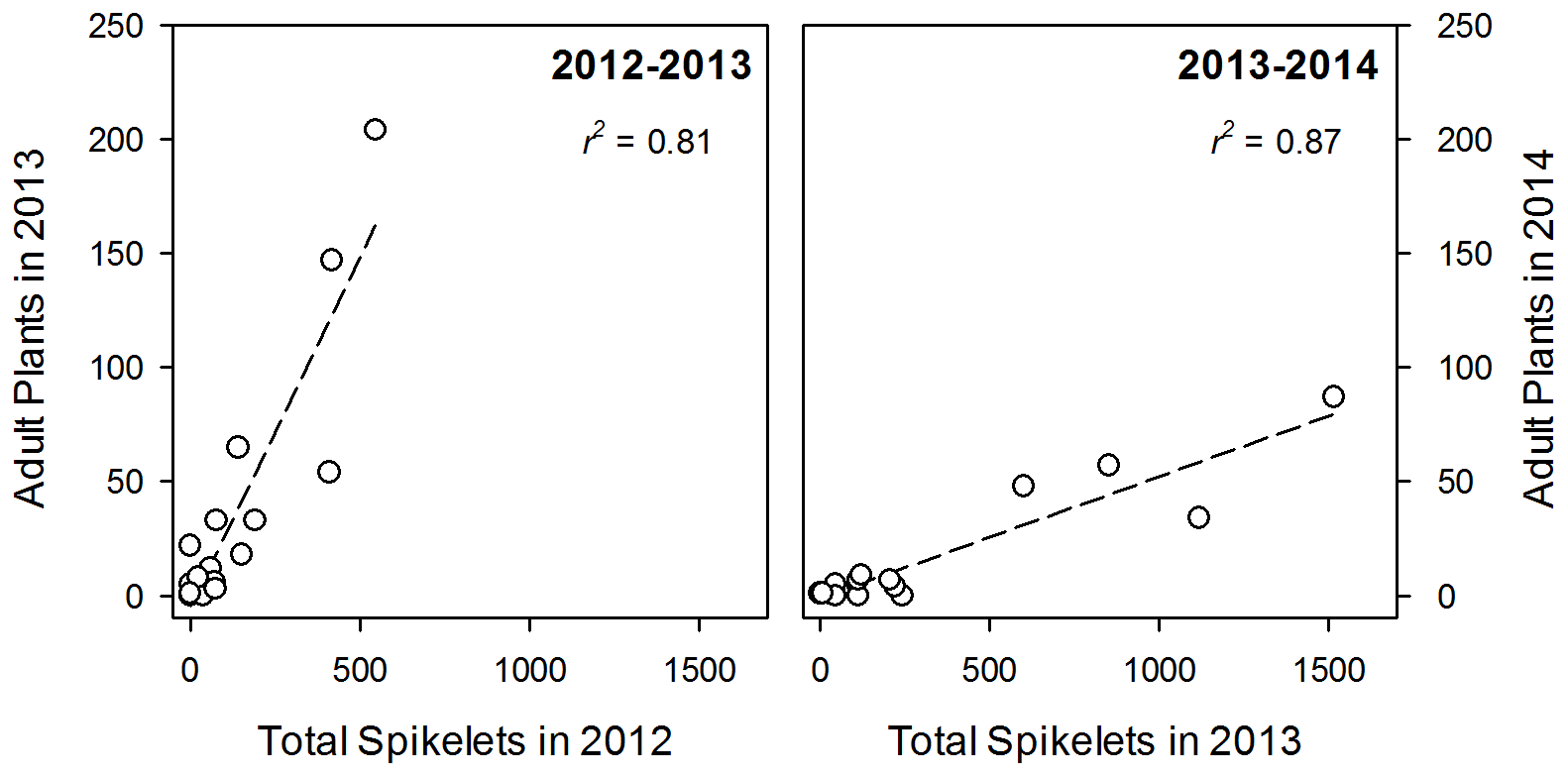


**Methods for LTRE (Life Table Response Experiment)**

Following Caswell (2001), the contributions to observed differences in λ*_seeds_* between experimentally disturbed versus control plots were calculated by multiplying the differences in mean transition values (“Disturbed” – “Control”) by the sensitivity values of their “Midway” (mean) transition values. Sensitivity values were determined by simply increasing each transition value by 10^-6^ to determine the effect on λ*_seeds_* (sensitivity value = Δ λ / Δ transition value).

All transition values were calculated as the mean of plot-level values, with 10,000 boot-strapped iterations. Confidence intervals (shown on Fig. 2D) were calculated to correct for bias (Caswell 2001).

**Table S1.** Transition and sensitivity values used in the LTRE to compare experimentally disturbed versus control plots.

**Transition Values**

|  | **Establishment** | **Survival** | **Fecundity** | **λ*_seeds_*** |
| --- | --- | --- | --- | --- |
| **Control** | 0.013 | 0.542 | 5.10 | 0.035 |
| **Disturbed** | 0.133 | 0.553 | 3.38 | 0.247 |

**Sensitivity Values**

|  | **Establishment** | **Survival** | **Fecundity** |
| --- | --- | --- | --- |
| **Control** | 2.763 | 0.064 | 0.007 |
| **Disturbed** | 1.866 | 0.447 | 0.073 |

**“Midway” values used for LTRE**

|  | **Establishment** | **Survival** | **Fecundity** |
| --- | --- | --- | --- |
| **Transition Values** | 0.073 | 0.547 | 4.248 |
| **Sensitivity Values** | 2.319 | 0.307 | 0.040 |

**LTRE**

|  | **Establishment** | **Survival** | **Fecundity** |
| --- | --- | --- | --- |
| **Disturbed - Control** | 0.120 | 0.011 | -1.723 |
| **Contribution*** | 0.278 | 0.003 | -0.068 |

*Contribution = (Disturbed - Control)×(Midway Sensitivity Value)

**Meteorological Data**

Temperature and precipitation data were obtained from the Cape Cod Remote Automatic Weather Station (RAWS; NWS ID: CAOM3, 41.975°N 70.025°W). Snow depth data were obtained from the National Operational Hydrologic Remote Sensing Center (NOHRSC; Station ID: Wellfleet 597WELL, 41.936°N 70.033°W). Degree days were calculated with a base of 0°C and were assumed to not accumulate during periods of snow cover.

**Table S2**. Accumulated degree days (°C) for each year assuming different starting dates. April 15 is used a benchmark based on the observation of seedlings that had just emerged on that date in 2013.

|  | **Accumulated Degree Days**  **On April 15** | | |  | **Difference in Days Compared to Accumulated**  **Degree Days on April 15 2013** | | |
| --- | --- | --- | --- | --- | --- | --- | --- |
| **Starting Date** | **2012** | **2013** | **2014** |  | **2012 vs. 2013** | **2012 vs. 2014** | **2014 vs. 2013** |
| 1-Sep | 1944.4 | 1623.4 | 1497.7 |  | -46 | -63 | 17 |
| 1-Oct | 1389.4 | 1086 | 976.3 |  | -39 | -55 | 16 |
| 1-Nov | 961.8 | 662 | 563.5 |  | -39 | -53 | 14 |
| 1-Dec | 661.2 | 450.8 | 363.3 |  | -26 | -39 | 13 |
| 1-Jan | 488.6 | 291.6 | 260.9 |  | -25 | -31 | 6 |

**Figure S2**. Daily precipitation, snow depth, and accumulated degree days starting on 1 December of each year. The dotted line indicates when cumulative degree days reached 450, which is when seedlings were observed to be emerging on 15 April 2013.


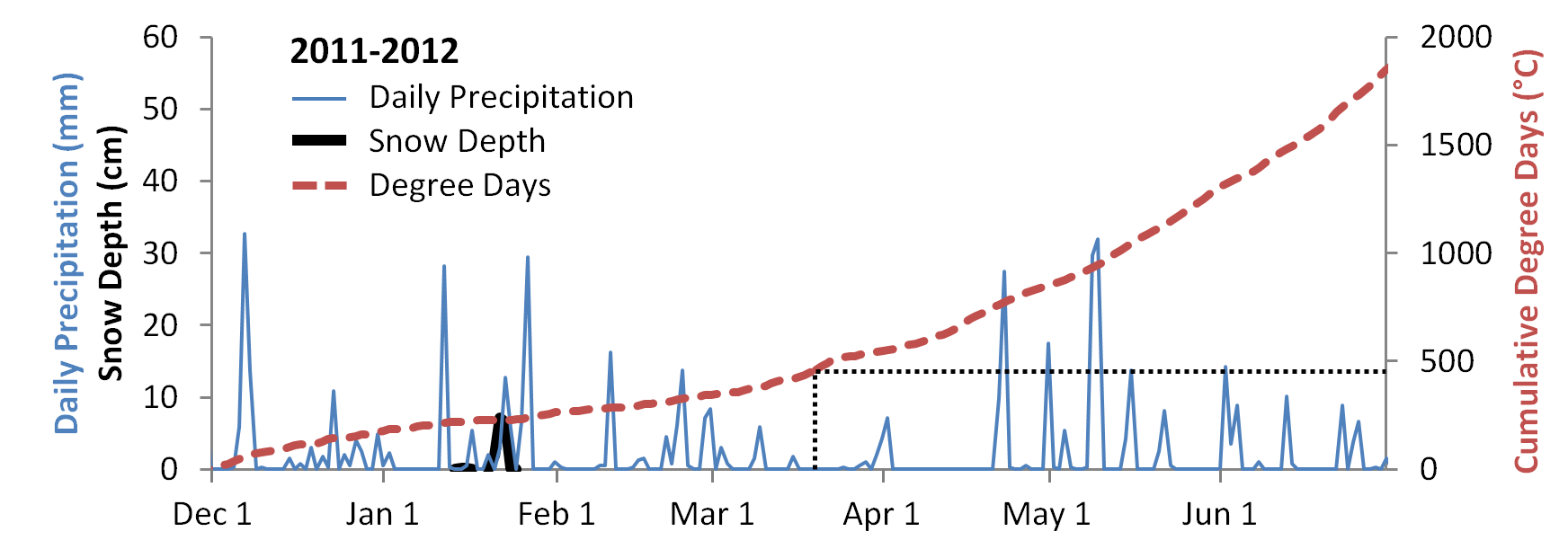


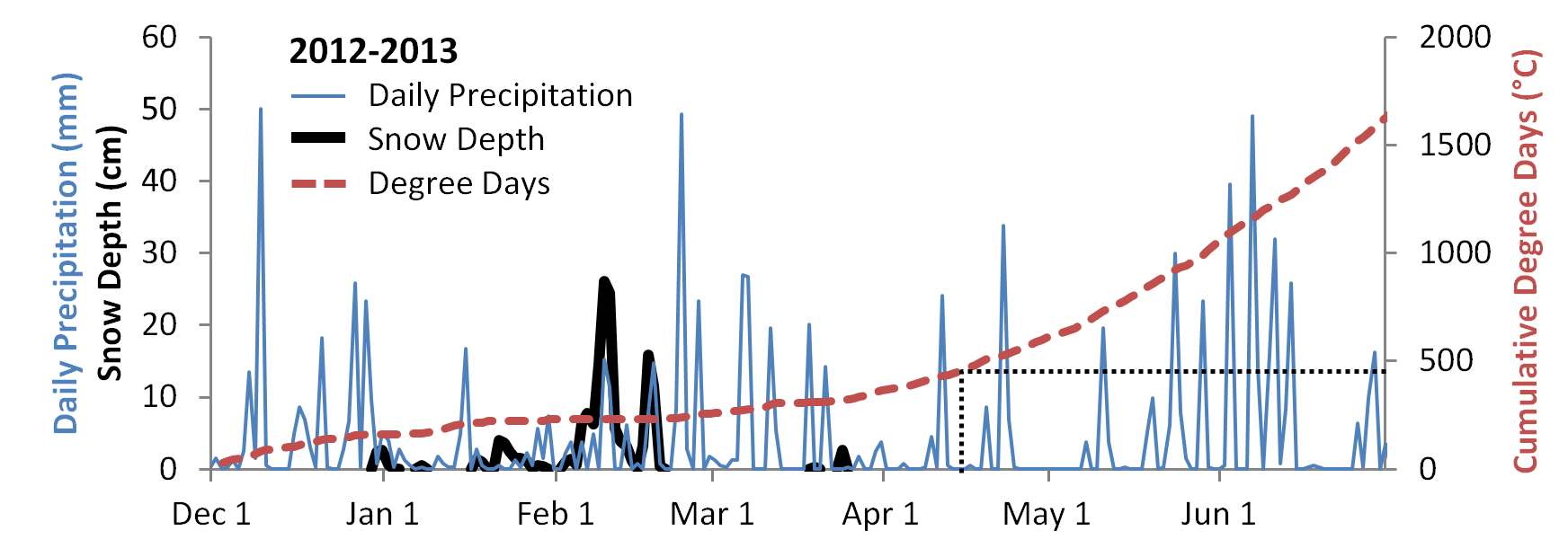


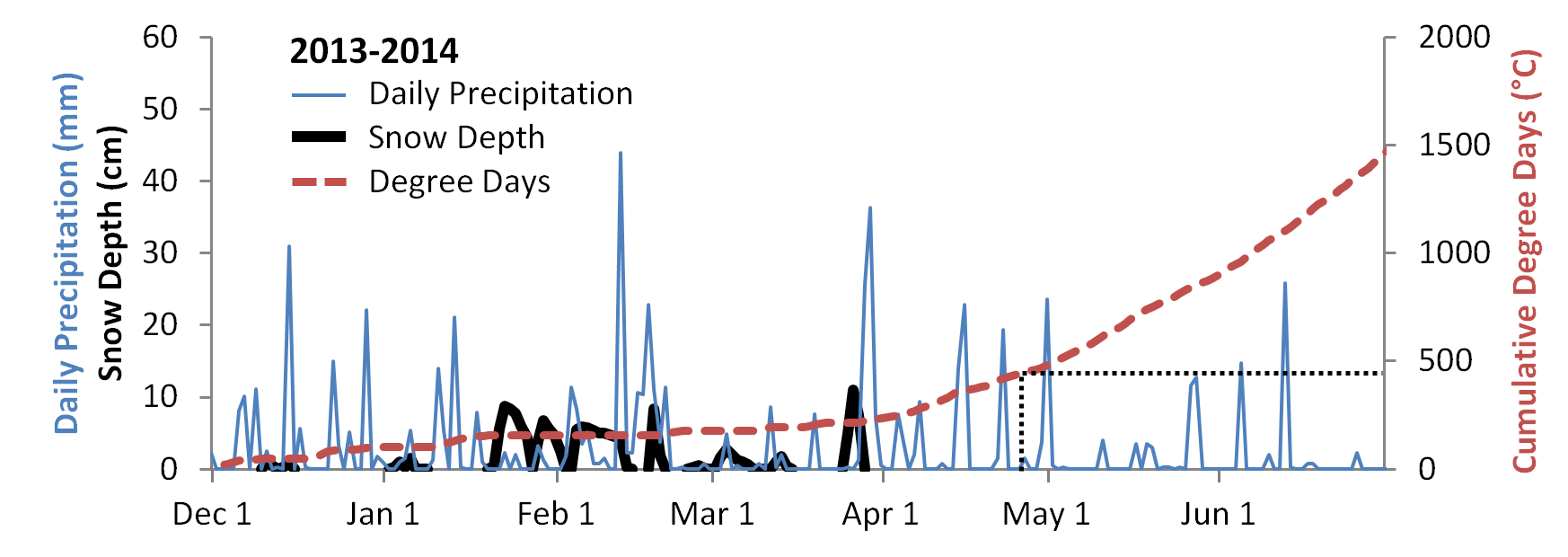


**LITERATURE CITED**

Caswell H. 2001. *Matrix population models: Construction, analysis, and interpretation.* Sunderland, Massachusetts: Sinauer Associates.
